# INTER-RATER RELIABILITY AND CONSTRUCT VALIDITY OF A CROSS-DIAGNOSTIC MOVEMENT QUALITY SCORE FOR REHABILITATION ASSESSMENT

**DOI:** 10.2340/jrm.v58.45701

**Published:** 2026-06-23

**Authors:** Koji OHATA, Takeshi KOZAKAI, Kazuhisa SOMEYA, Takatomi NARITA, Natsuki WATANABE, Yuko MINE, Shinichi DAIKUYA

**Affiliations:** 1 Department of Physical Therapy, Hokuriku University, Kanazawa; 2 Hokuriku Hospital, Kanazawa; 3 Kasumigaseki-Minami Hospital, Kawagoe; 4 Nishinomiya Kyoritsu Rehabilitation Hospital, Nishinomiya; 5 Utsunomiya Memorial Hospital, Utsunomiya; 6 Department of Medical Humanities, School of Medicine, University of Occupational and Environmental Health, Kitakyushu, Japan

**Keywords:** movement quality, physical therapy assessment, inter-rater reliability, construct validity, functional independence measure, rehabilitation

## Abstract

**Objective:**

To examine the inter-rater reliability and construct validity of a Movement Quality Score (MQS) within the Standard Physical Therapy Assessment (SPTA) across diagnostic groups.

**Design:**

Cross-sectional study.

**Subjects and Methods:**

Eighty-four hospitalized patients undergoing rehabilitation for neurological, musculoskeletal, or internal medical conditions were included. Inter-rater reliability was assessed in 68 participants independently evaluated by 2 physiotherapists. The MQS was scored using the SPTA component. Inter-rater reliability of the summed score was analysed using the intraclass correlation coefficient (ICC [2,1]). Construct validity was examined using Spearman’s rank correlations between the MQS and the Functional Independence Measure (FIM) motor total score and its subdomains (self-care, sphincter control, transfers, and locomotion), as well as other SPTA components.

**Results:**

The MQS demonstrated excellent inter-rater reliability (ICC [2,1] = 0.93). It showed a very strong correlation with the FIM motor total score (ρ = 0.91, *p* < 0.001), with strong associations for transfers and locomotion (ρ = 0.92) and self-care (ρ = 0.86). The correlation with sphincter control was lower (ρ = 0.68).

**Conclusion:**

The MQS demonstrates excellent reliability and strong construct validity, supporting its use as a movement-focused complement to activities of daily living-based assessments in heterogeneous rehabilitation populations.

## LAY ABSTRACT

This study examined whether a new assessment, the Movement Quality Score, can reliably evaluate how well patients perform basic movements during rehabilitation. We tested the Movement Quality Score in hospitalized patients with neurological, musculoskeletal, and internal medical conditions. The score showed excellent agreement between different physiotherapists and was strongly related to established measures of daily function. These findings suggest that the Movement Quality Score may be useful as a complementary tool for assessing movement quality across different rehabilitation populations.

Developing reliable and valid clinical assessments remains a key issue in rehabilitation medicine. The scope of rehabilitation has expanded beyond traditional musculoskeletal and neurological disorders to include cardiovascular and respiratory diseases, cancer, other internal medical conditions, and frailty in older adults (1–4). Recent international initiatives have emphasized rehabilitation as an essential health service across health conditions and across the life course, highlighting the need for information systems and standardized assessment approaches that can capture functioning across diverse populations (1, 2). Although increasing specialization has enhanced disease-specific expertise, it has also highlighted the need for standardized, cross-diagnostic assessment tools that can evaluate functioning consistently across diverse patient populations, facilitate interdisciplinary communication, and improve outcome comparability.

The World Health Organization addressed this issue through the International Classification of Functioning, Disability and Health (ICF), which defines functioning as the interaction among body functions and structures, activities, participation, and contextual factors (5). From the ICF perspective, clinical assessments can be broadly categorized into impairment-based assessments, which focus on deficits in body functions and structures, and activity-based assessments, which evaluate limitations in activity execution (6).

Many existing assessments are impairment-based, focusing on deficits associated with specific symptoms and thus often reflecting disease-specific characteristics. In contrast, activity-based assessments are generally applicable across a range of conditions but may be more influenced by contextual and environmental factors.

Within the ICF, the activity domain is further divided into capacity and performance. Capacity reflects an individual’s ability to execute a task under standardized or controlled conditions, whereas performance represents the execution of that task in real-life contexts (7). In clinical rehabilitation, many widely used activity-based measures, particularly independence scales, are primarily outcome-focused, emphasizing task accomplishment or the level of assistance required, and thus predominantly reflect performance. For example, the Functional Independence Measure (FIM) evaluates independence based on the amount of assistance required to perform daily activities, reflecting performance in everyday contexts rather than the quality of movement itself. This perspective is consistent with previous work (7) showing that the FIM aligns more closely with ICF performance constructs than with standardized capacity measures. Consequently, qualitative aspects of motor execution are not explicitly captured in such assessments.

To address this conceptual limitation and grounded in the ICF distinction between capacity and performance, we developed a Movement Quality Score (MQS) to incorporate the evaluation of movement quality during activity execution. The score assesses fundamental motor tasks, including rolling, sitting up, sit-to-stand, sitting and standing balance, walking, and stair negotiation. These tasks represent core components of physical therapy assessment and are therefore core skills routinely assessed in clinical practice. By integrating evaluation of both task execution and qualitative movement characteristics, this approach enables a more comprehensive activity-based assessment of motor function within the ICF framework.

The comparison with the FIM was used to examine construct validity because the FIM is a widely used activities of daily living (ADL)-based performance measure, whereas the MQS is intended to capture qualitative aspects of movement execution during fundamental motor tasks. Therefore, this comparison was not intended to show that the 2 measures assess the same construct, but rather to determine whether the MQS is related to, and conceptually distinct from, conventional independence-based measures of activity performance.

The purpose of this study was to develop this ICF-based, cross-diagnostic score and to evaluate its inter-rater reliability and construct validity through comparison with the Functional Independence Measure in a heterogeneous population of patients undergoing rehabilitation.

## METHODS

### Participants

Participants were conveniently recruited from inpatients who were receiving rehabilitation at the participating hospitals and were considered eligible for assessment by the local investigators or research collaborators. The participating facilities included Hokuriku Hospital (Kanazawa, Ishikawa, Japan), Kasumigaseki-Minami Hospital (Kawagoe, Saitama, Japan), Nishinomiya Kyoritsu Rehabilitation Hospital (Nishinomiya, Hyogo, Japan), and Utsunomiya Memorial Hospital (Utsunomiya, Tochigi, Japan). According to the original study protocol, the study planned to recruit approximately 20 participants from each participating facility, including the institutions of the co-investigators and research collaborators affiliated with the Japanese Physical Therapy Association.

The inclusion criteria were as follows: (*i*) hospitalized patients in the acute or subacute phase; (*ii*) patients receiving rehabilitation for neurological, musculoskeletal, or internal medical conditions; (*iii*) patients who were able to undergo the SPTA/MQS and FIM assessments; and (*iv*) patients who provided written informed consent. No additional formal exclusion criteria were specified in the study protocol. However, patients who could not undergo the SPTA/MQS or FIM assessment or who did not provide informed consent were not enrolled.

Patients were enrolled only after receiving verbal and written explanations of the study from a co-investigator or research collaborator and providing consent both verbally and in writing. A total of 84 patients with neurological, musculoskeletal, or internal medical conditions were enrolled. The distribution of clinical diagnoses is summarized in Table I. Fifty-nine licensed physiotherapists employed at the participating hospitals served as examiners. For the analysis of inter-rater reliability, 68 patients were independently assessed by 2 physiotherapists. The remaining 16 patients were included in the construct validity analysis but were not included in the inter-rater reliability analysis because a second independent rating could not be completed due to time constraints. Data were collected between October 12 and November 16, 2020. All data were anonymized within each participating facility before analysis, and no personally identifiable information linked to individual participants was transferred outside the participating facilities. The study protocol was approved by the Ethics Committee of the Japanese Society of Physical Therapy (Approval number: R02-005).

**Table I:** Participant characteristics

Clinical category	*n*	Main diagnoses
Neurological disorders	21	Intracerebral haemorrhage, ischaemic stroke, subarachnoid haemorrhage, Parkinson’s disease
Musculoskeletal disorders	46	Femoral fracture, osteoarthritis of the knee, anterior cruciate ligament injury, lumbar spinal stenosis
Internal medical conditions	17	Aortic stenosis, postoperative ileus, bacterial pneumonia, congestive heart failure, chronic kidney disease

### Study design and assessment procedures

This was a cross-sectional study designed to examine the reliability and validity of the newly developed assessment. The assessment was administered during routine rehabilitation sessions. For the analysis of inter-rater reliability, 2 physiotherapists independently evaluated the same patients within a short interval (approximately 2–3 days), during which no significant clinical change was observed. The raters were blinded to each other’s scores.

Construct validity was examined by comparing the total score of the new assessment with the motor subscale of the Functional Independence Measure. The FIM assessment was conducted on the same day as the new assessment by 1 of the physical therapists who performed the new evaluation.

### Outcome measures

*Movement Quality Score (MQS).* The Movement Quality Score (MQS) constitutes a core component of the Standard Physical Therapy Assessment (SPTA), developed by the Standardized Assessment Development Committee of the Japanese Physical Therapy Association. The SPTA is a comprehensive standardized assessment framework in physical therapy; the present study focuses specifically on its movement quality component. A study-specific English summary of the MQS and related SPTA scoring items analysed in this study, based on the original Japanese assessment form (Ver. 2.1), is provided as Appendix S1.

The MQS consists of 10 items designed to evaluate fundamental movement abilities across key domains of physical therapy practice: rolling, sitting up, sitting balance, sit-to-stand, standing balance, walking independence, walking speed, gait abnormality, and stair negotiation. Core movement tasks are rated on a 5-point ordinal scale, standing balance items on a 3-point ordinal scale, walking independence on a 6-point ordinal scale, walking speed and gait abnormality on 3-point ordinal scales, and stair negotiation on a 4-point ordinal scale. Higher scores indicate better movement performance or fewer movement abnormalities. The summed MQS was used for reliability and validity analyses.

*Other SPTA components.* In addition to the MQS, the SPTA includes impairment-based assessments and activity-based assessments. In the present study, muscle strength and pain were analysed as impairment-based components, whereas upper extremity function and life-space mobility were analysed as activity-based components.

Muscle strength was assessed for ankle dorsiflexion, knee extension, and hip flexion using a 4-level ordinal scale based on manual muscle testing with reference to the Medical Research Council scale (10). Pain was evaluated separately at rest and during movement using a 3-level ordinal scale. Upper extremity function was assessed with reference to the Motor Activity Log (11), based on the quality and frequency of object manipulation in a selected daily activity. Life-space mobility was assessed using the Japanese version of the Life-Space Assessment (LSA-J) (12), and analyses were limited to mobility within the home and around the home. Detailed scoring criteria for these SPTA components are provided in Appendix S1.

*Functional Independence Measure (FIM).* The Functional Independence Measure (FIM) is a widely used standardized instrument assessing functional status across diverse patient populations, with established reliability and reproducibility (13). The FIM comprises 18 items in 2 domains: a motor domain and a cognitive domain. Each item is scored on a 7-point ordinal scale, with higher scores indicating greater functional independence.

In this study, analyses focused on the FIM motor domain. The motor score was analysed as the total motor score and as 3 subdomains: self-care, sphincter control, and transfers and locomotion. These subdomains were used to examine the relationship between the MQS and ADL-based functional independence.

### Statistical analysis

Statistical analyses were performed using SPSS (version 29.0; IBM Corp, Armonk, NY, USA). Inter-rater agreement for each SPTA item (the MQS and other items) was evaluated using weighted kappa coefficients with quadratic weights. For summed scores (the MQS, muscle strength, pain, upper limb function, and Life-Space Assessment [LSA]), inter-rater reliability was assessed using the intraclass correlation coefficient based on a 2-way random-effects model with absolute agreement for single measurements (ICC [2,1]). To explore disease-specific reliability, ICC analyses were additionally conducted separately for participants with neurological disorders, musculoskeletal disorders, and internal medical conditions.

The strength of agreement for both weighted kappa coefficients and ICC values was interpreted as follows: poor (< 0.50), moderate (0.50–0.74), good (0.75–0.89), and excellent (≥ 0.90) (14). Absolute reliability was examined by calculating the standard error of measurement (SEM) and the minimal detectable change at the 95% confidence level (MDC_95_), according to previously established methods for quantifying measurement error and interpretability of change scores (15). In addition, the SEM percentage (SEM_%range_) was calculated by dividing the SEM by the maximum possible score of the scale and multiplying by 100, to express measurement error relative to the scale range.

Construct validity of the MQS was examined based on the framework of the International Classification of Functioning, Disability and Health (ICF). It was hypothesized that the MQS would demonstrate very strong to strong correlations with ADL measures, moderate to strong correlations with body function measures, and weaker correlations with pain.

Spearman’s rank correlation coefficients were calculated between the MQS and the following clinical measures: the Functional Independence Measure (FIM) motor total score and its 3 subdomains (self-care, sphincter control, and transfers and locomotion) representing ADL function; muscle strength and pain representing body function; and upper extremity (UE) function and life-space assessment (LSA) representing activity.

Spearman’s correlation was selected due to the ordinal nature of the scales and potential deviations from normal distribution. Correlation strength was interpreted based on commonly used criteria as negligible (< 0.30), weak (0.30–0.49), moderate (0.50–0.69), strong (0.70–0.89), and very strong (≥ 0.90). Statistical significance was set at *p* < 0.05. Correlation coefficients (ρ) with 95% confidence intervals (CIs) and *p*-values were reported.

## RESULTS

A total of 84 patients were enrolled and included in the construct validity analysis. Among them, 68 patients were independently assessed by two physiotherapists and were included in the inter-rater reliability analysis. The remaining 16 patients were not included in the inter-rater reliability analysis because a second independent rating could not be completed due to time constraints, but they were retained in the construct validity analysis. Due to a recording error in the muscle strength data of one participant, analyses involving muscle strength were conducted in 67 patients for reliability and 83 patients for validity.

## INTER-RATER RELIABILITY

*Item-level agreement.* Quadratic weighted κ coefficients for individual MQS items ranged from 0.65 to 0.91 (Table II). Agreement was excellent for walking independence (κ = 0.91), good for sitting up, sitting balance, sit-to-stand, walking speed, and stair negotiation (κ = 0.77–0.86), and moderate for rolling, standing balance, and gait abnormality (κ = 0.65–0.72).

**Table II:** Quadratic weighted κ of each item in SPTA

Items	Quadratic weighted κ	95% CI	Percentage agreement (%)	Interpretation
SPTA				
1. Rolling	0.70	0.54–0.86	63.2	Moderate
2. Sitting up	0.79	0.67–0.91	67.6	Good
3. Sitting balance	0.80	0.65–0.95	79.4	Good
4. Sit-to-stand	0.77	0.64–0.90	63.2	Good
5. Standing balance: reaching	0.72	0.58–0.85	66.2	Moderate
6. Standing balance: tandem	0.65	0.48–0.82	67.6	Moderate
7. Walking: independence	0.91	0.86–0.96	66.2	Excellent
8. Walking: speed	0.86	0.77–0.96	83.8	Good
9. Walking: abnormality	0.68	0.50–0.86	75.0	Moderate
10. Stair negotiation	0.82	0.72–0.92	69.1	Good
Other measure				
Muscle strength: Ankle DF	0.81	0.72–0.90	73.1	Good
Muscle strength: Knee extension	0.71	0.54–0.88	77.6	Moderate
Muscle strength: Hip flexion	0.71	0.54–0.88	77.6	Moderate
Pain: rest	0.25	–0.08–0.58	72.1	Poor
Pain: movement	0.55	0.35–0.75	76.5	Moderate
UE function: Quality	0.71	0.58–0.84	47.8	Moderate
UE function: Frequency	0.67	0.50–0.84	73.5	Moderate
LSA: within the home	0.44	0.20–0.68	54.4	Poor
LSA: around the home	0.55	0.29–0.80	70.1	Moderate

Among the other SPTA components, muscle strength items demonstrated moderate to good agreement. Ankle dorsiflexion showed good agreement (κ = 0.81), while knee extension and hip flexion showed moderate agreement (κ = 0.71). Pain during movement showed moderate agreement (κ = 0.55), whereas pain at rest demonstrated poor agreement (κ = 0.25). Upper extremity function items showed moderate agreement (κ = 0.67–0.71), and life-space mobility ranged from poor to moderate (κ = 0.44–0.55).

*Reliability of summed scores.* The total MQS demonstrated excellent inter-rater reliability (ICC(2,1) = 0.93, 95% CI 0.89–0.96), with an SEM of 2.5 and an MDC_95_ of 6.9 (Table III). Disease-specific analyses revealed excellent reliability in the neurological and musculoskeletal groups, and good reliability in the internal medical group.

**Table III:** Inter-rater reliability and absolute reliability of SPTA scores

Variable	Group	ICC (2,1)	95% CI	Interpretation	SEM	SEM%_range_	MDC_95_
MQS	Total	0.93	0.89–0.96	Excellent	2.5	7.8	6.9
	Neurological	0.90	0.75–0.96	Excellent	3.0	9.4	8.2
	Musculoskeletal	0.95	0.91–0.98	Excellent	2.0	6.2	5.5
	Internal	0.89	0.71–0.96	Good	2.9	9.2	8.1
Muscle strength	Total	0.84	0.76–0.90	Good	0.8	8.5	2.1
	Neurological	0.85	0.62–0.94	Good	0.9	10.4	2.6
	Musculoskeletal	0.91	0.83–0.95	Excellent	0.6	7.0	1.7
	Internal	0.44	–0.10–0.77	Poor	0.9	9.5	2.4
Pain	Total	0.37	0.15–0.56	Poor	0.7	16.4	1.8
	Neurological	0.43	–0.59–0.75	Poor	0.4	9.7	1.1
	Musculoskeletal	0.17	–0.17–0.64	Poor	0.7	17.0	1.9
	Internal	0.37	–0.13–0.73	Poor	0.7	17.5	1.9
UE function	Total	0.78	0.67–0.86	Good	0.9	13.3	2.6
	Neurological	0.68	0.30–0.87	Moderate	1.1	16.1	3.1
	Musculoskeletal	0.83	0.70–0.91	Good	0.8	11.0	2.1
	Internal	0.70	0.20–0.90	Moderate	1.0	13.6	2.6
LSA	Total	0.60	0.42–0.73	Moderate	1.8	15.1	5.0
	Neurological	0.69	0.32–0.88	Moderate	1.5	12.8	4.3
	Musculoskeletal	0.60	0.34–0.77	Moderate	2.0	16.8	5.6
	Internal	0.23	–0.27–0.65	Poor	1.4	11.8	3.9

Summed muscle strength scores demonstrated good reliability in total (ICC = 0.84, 95% CI 0.76–0.90), with excellent reliability in the musculoskeletal subgroup, good reliability in the neurological subgroup, and poor reliability in the internal medical subgroup. Summed pain scores showed poor reliability (ICC = 0.37). Upper extremity function scores were reliable overall (ICC = 0.78), with moderate to good reliability across subgroups, whereas life-space mobility demonstrated moderate reliability (ICC = 0.60).

### Construct validity

*Correlation with ADL measures.* The MQS showed a very strong positive correlation with the FIM motor total score (ρ = 0.91, 95% CI 0.86–0.94, *p* < 0.001; Table IV).

**Table IV:** Correlation of the MQS with ICF-related measures

Category	Variables	*n*	ρ	95% CI	*p*-value
ADL function	FIM motor total	84	0.91	0.86–0.94	< 0.001
	Self-care	84	0.86	0.79–0.91	< 0.001
	Sphincter control	84	0.68	0.54–0.78	< 0.001
	Transfers & locomotion	84	0.92	0.87–0.95	< 0.001
Impairment-based	Muscle strength	83	0.75	0.63–0.83	< 0.001
	Pain	84	0.22	–0.02–0.44	0.068
Activity-based	UE function	84	0.59	0.41–0.73	< 0.001
	LSA	84	0.51	0.30–0.67	< 0.001

Very strong correlations were observed with transfers and locomotion (ρ = 0.92), and strong correlations with self-care (ρ = 0.86), whereas sphincter control showed a moderate correlation (ρ = 0.68).

**Fig. 1:**
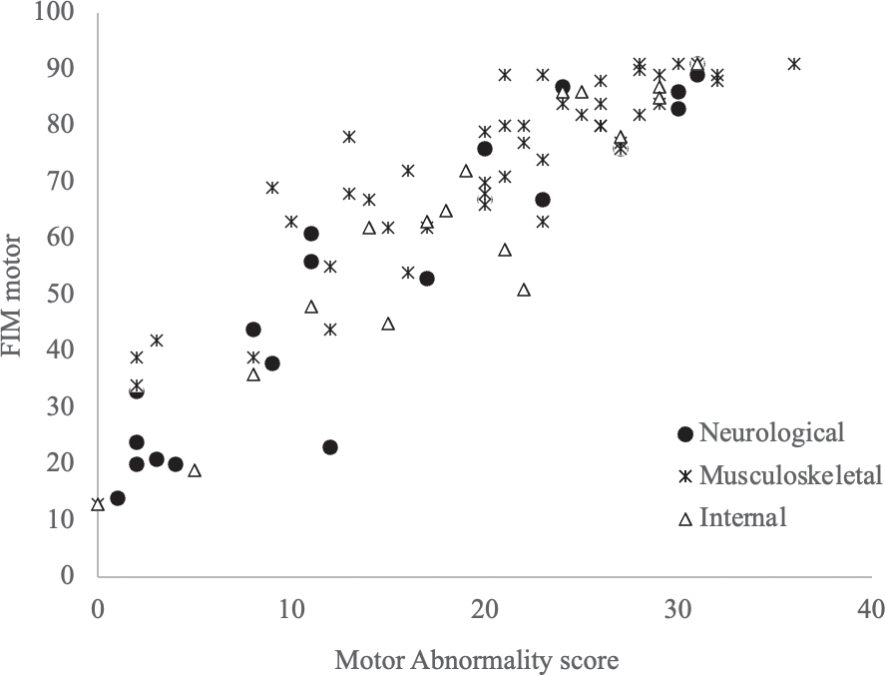
Relationship between MQS and FIM motor score.

Fig. 1 illustrates the relationship between the MQS score and the FIM motor score. When analysed by clinical category, very strong correlations were observed in the neurological group (ρ = 0.93, 95% CI 0.82–0.97, *p* < 0.001), musculoskeletal group (ρ = 0.87, 95% CI 0.77–0.93, *p* < 0.001), and internal medical group (ρ = 0.90, 95% CI 0.74–0.97, *p* < 0.001).

*Correlation with impairment-based measures.* The MQS demonstrated a strong positive correlation with muscle strength (ρ = 0.75, 95% CI 0.63–0.83, *p* < 0.001). In contrast, the correlation with pain was weak and did not reach statistical significance (ρ = 0.22, 95% CI −0.02–0.44, *p* = 0.068).

*Correlation with activity-based measures.* A moderate positive correlation was observed between the MQS and upper extremity function (ρ = 0.59, 95% CI 0.41–0.73, *p* < 0.001). Life-space mobility was also moderately correlated with the MQS (ρ = 0.51, 95% CI 0.30–0.67, *p* < 0.001).

Overall, the MQS showed moderate to strong associations with activity-based measures. In contrast, within impairment-based measures, it showed a strong association with muscle strength but only a weak and non-significant association with pain.

## DISCUSSION

The present study examined the inter-rater reliability and construct validity of the movement quality component of the SPTA, an activity-based assessment designed to evaluate qualitative movement execution rather than independence alone, in a heterogeneous acute and sub-acute rehabilitation population. Although both the MQS and the FIM are situated within the ICF activity domain, they differ conceptually in focus: the FIM primarily quantifies independence and assistance requirements in real-life contexts, whereas the MQS emphasizes observable movement deviations during structured fundamental motor tasks. Thus, while sharing the same activity-level framework as the FIM, the MQS provides complementary information regarding movement quality that is not directly captured by independence-based measures.

### Reliability of the MQS

The MQS demonstrated excellent inter-rater reliability (ICC = 0.93), with consistently high values across diagnostic subgroups. The SEM and MDC_95_ further supported the clinical applicability of the scale by providing indices of measurement precision and thresholds for detecting meaningful change.

The MQS evaluates observable deviations during structured fundamental motor tasks, anchoring scoring to visible and standardized task execution. By assessing both execution (performance) and capability (capacity), the score aligns with the activity construct of the ICF, capturing how a task is performed under controlled conditions as well as in real-life contexts. This dual perspective likely contributes to the high reliability by reducing variability due to task complexity or environmental factors, providing a more stable indicator of motor function across heterogeneous patient populations.

These findings are consistent with previous reports demonstrating high inter-rater reliability for performance-based observational measures, such as the FIM (16) and the Berg Balance Scale (BBS) (17), which rely on structured observation of task performance and clearly operationalized scoring criteria.

### Reliability of the other components

The summed muscle strength score, an impairment-based measure, demonstrated good reliability overall (ICC = 0.84), with excellent reliability in the musculoskeletal subgroup (ICC = 0.91) and good reliability in the neurological subgroup (ICC = 0.85). These results are consistent with previous reports showing high inter-rater reliability for the Medical Research Council (MRC) sum score under standardized conditions, particularly in these populations (18, 19). Reliability was lower in the internal medical subgroup (ICC = 0.44), suggesting that while standardized measurement protocols generally yield high reliability, disease-specific factors can influence consistency. In contrast, pain, another impairment-based measure, showed weak and non-significant reliability (ρ = 0.22, *p* = 0.068) across subgroups, indicating that symptom-level variables may fluctuate independently of underlying pathology and are thus less stable for standardized assessment.

Upper extremity function, an activity-based measure, demonstrated good reliability overall (ICC = 0.78). Although the original Motor Activity Log (MAL) has shown excellent reliability in stroke populations (ICC 0.87–0.95) (20), the slightly lower ICC observed here may reflect the broader diagnostic spectrum and differences in movement characteristics across conditions, while remaining acceptable for clinical use. Life-space mobility (LSA), also an activity-based measure, demonstrated moderate reliability overall (ICC = 0.60). Previous studies in community-dwelling older adults reported higher reliability (ICC 0.74–0.96) (21,22), whereas in our heterogeneous inpatient sample, environmental and contextual factors, such as hospitalization and limited mobility opportunities, may have influenced scores.

These findings highlight that impairment-based measures are often susceptible to fluctuations related to disease-specific symptoms, while activity-based measures can also be affected by environmental and contextual factors. In contrast, movement quality-focused activity measures, such as the MQS, provide a more stable and broadly applicable approach for evaluating motor function across diverse rehabilitation populations.

### Construct validity

Construct validity findings were consistent with predefined hypotheses derived from the ICF framework, considering both impairment-based and activity-based constructs. As the primary reference for activity-based validation, the MQS demonstrated a very strong correlation with the FIM motor total score (ρ = 0.91), particularly with Transfers & locomotion (ρ = 0.92) and Self-care (ρ = 0.86), indicating convergence with performance-oriented activity measures. The association with Sphincter control was comparatively lower (ρ = 0.68), which is theoretically plausible because continence primarily reflects autonomic function rather than qualitative movement performance.

High correlations were observed across all diagnostic subgroups (ρ = 0.87–0.93), indicating that the MQS provides a consistent activity-based evaluation regardless of disease category. Importantly, despite assessing aspects of movement quality not directly captured by performance outcome measures such as the FIM, the MQS still showed strong convergence, suggesting that qualitative movement characteristics substantially influence performance outcomes in daily activities.

A strong association with muscle strength (ρ = 0.75) indicates convergence with relevant impairment-based constructs, whereas moderate correlations with upper extremity function (ρ = 0.59) and life-space mobility (ρ = 0.51) further support the scale’s validity across broader activity-level domains. The weak and non-significant correlation with pain (ρ = 0.22, *p* = 0.068) reinforces construct differentiation, showing that symptom-level variables are distinct from both impairment- and activity-based measures.

Overall, this pattern of correlations – stronger relationships with movement-related constructs across both impairment- and activity-based measures, and weaker relationships with symptom-level and continence-related variables – supports the scale’s construct coherence within the ICF framework.

### Clinical implications

The MQS provides a reliable and clinically meaningful indicator of movement quality across diagnostic categories. By focusing on qualitative aspects of task execution within an activity-based framework, rather than solely on independence or impairment-level measures, it complements existing ADL-based and performance outcome measures. This approach may help identify specific movement deviations that are amenable to therapeutic intervention and support movement-focused rehabilitation planning across diverse patient populations.

### Limitations

Several limitations should be acknowledged. First, the cross-sectional design precluded evaluation of responsiveness. Second, recruitment from hospital-based rehabilitation settings may limit generalizability. Third, raters were not blinded to patients’ clinical presentation, although they were blinded to each other’s scores. Fourth, demographic variables were not recorded to minimize personal data collection, potentially limiting external validity. Finally, some subgroup analyses involved small samples, particularly in the internal medical group, which may contribute to the variability observed in reliability estimates for this subgroup.

### Conclusion

In conclusion, the MQS component of the SPTA demonstrated excellent inter-rater reliability and construct validity in a heterogeneous acute and sub-acute rehabilitation population. The scale showed strong convergence with both impairment-based and activity-based measures while maintaining discriminant validity from symptom-level and continence-related variables, consistent with hypotheses derived from the ICF framework. Compared with conventional impairment-based or context-dependent assessments, a movement quality-focused activity measure may provide a more stable and broadly applicable indicator across diverse diagnostic conditions. Although reliability of other components varied according to clinical subgroup and patient stability, the MQS maintained robust measurement properties. These findings support its potential utility as a movement quality-focused activity assessment capable of complementing conventional ADL measures by clarifying how patients perform movements, not only whether they can perform activities independently. The MQS may therefore contribute to more detailed rehabilitation assessment, goal setting, and movement-focused treatment planning in heterogeneous clinical settings.

## Supplementary Material


