# Supplementary material for: INTER-RATER RELIABILITY AND CONSTRUCT VALIDITY OF A CROSS-DIAGNOSTIC MOVEMENT QUALITY SCORE FOR REHABILITATION ASSESSMENT
Source: J Rehabil Med. 2026 Jun 23;58:45701. doi: 10.2340/jrm.v58.45701 (PMC13307465; doi:10.2340/jrm.v58.45701)
Supplement: Supplementary file 1 [file JRM-58-45701-s1.pdf]

Appendix S1

Movement Quality Score and Related Standard Physical Therapy Assessment Scoring Sheet

Purpose and scope. This appendix provides an English, study-specific summary of the Movement Quality Score (MQS) and related components of the Standard Physical Therapy Assessment (SPTA) analyzed in the present study. It is based on the original Japanese assessment form (Ver. 2.1) developed by the Standardized Assessment Development Committee of the Japanese Physical Therapy Association. Items not analyzed in the present study, such as environmental assessment items and motivational assessment items, are not included.

General scoring instruction. For each item, the rater selects the single score that best describes the patient's status at the time of assessment. For core movement items, when the patient has the motor or activity capacity to perform the task but the score is reduced for reasons other than motor ability, the reason for score reduction may be recorded as: 1 = pain; 2 = medical management; 3 = fatigue; 4 = other. In this appendix, X indicates unable to assess or unknown when a study record cannot be classified.

Abbreviations. ADL, activities of daily living; LSA, Life-Space Assessment; MQS, Movement Quality Score; SPTA, Standard Physical Therapy Assessment; UE, upper extremity.

A. Movement Quality Score (MQS)

Domain	Item	Score	Scoring criteria
Core movement tasks	1. Rolling	0-4	4 = Performs normally in daily life without compensatory movement.<br>3 = Performs in daily life but with abnormal or compensatory movement.<br>2 = Can perform normally under assessment conditions, although not performed independently in daily life.<br>1 = Can perform under assessment conditions but with abnormal or compensatory movement.<br>0 = Unable to perform without assistance.<br>Note: The more difficult direction is assessed.
Core movement tasks	2. Sitting up	0-4	4 = Performs normally in daily life without compensatory movement.<br>3 = Performs in daily life but with abnormal or compensatory movement.<br>2 = Can perform normally under assessment conditions, although not performed independently in daily life.<br>1 = Can perform under assessment conditions but with abnormal or compensatory movement.<br>0 = Unable to perform without assistance.
Core movement tasks	3. Sitting balance	0-4	4 = Performs normally in daily life without compensatory movement.<br>3 = Performs in daily life but with abnormal or compensatory movement.<br>2 = Can perform normally under assessment conditions, although not performed independently in daily life.

			1 = Can perform under assessment conditions but with abnormal or compensatory movement.<br>0 = Unable to perform without assistance.<br>Note: Sitting unsupported at the edge of the bed for at least 30 seconds.
Core movement tasks	4. Sit-to-stand	0-4	4 = Performs normally in daily life without compensatory movement.<br>3 = Performs in daily life but with abnormal or compensatory movement.<br>2 = Can perform normally under assessment conditions, although not performed independently in daily life.<br>1 = Can perform under assessment conditions but with abnormal or compensatory movement.<br>0 = Unable to perform without assistance.
Standing balance	5. Forward reach toward the floor	0-2	2 = Can reach the floor with either hand and return to the starting position.<br>1 = Can reach below knee level with either hand and return to the starting position.<br>0 = Cannot reach below knee level with either hand.<br>Note: Knee flexion is permitted.
Standing balance	6. Tandem stance	0-2	2 = Can maintain balance with one heel placed directly in front of the other foot in a heel-to-toe position on both sides.<br>1 = Can maintain balance when one heel is placed in front of the other foot with approximately one foot-width between the feet; either side is acceptable.<br>0 = Cannot maintain balance when one heel is placed in front of the other foot.
Gait	7. Walking independence	0-5	5 = Independently walks on uneven surfaces, stairs, and slopes.<br>4 = Independently walks on level ground.<br>3 = Requires verbal cueing or supervision.<br>2 = Requires intermittent or continuous light-touch assistance.<br>1 = Requires continuous assistance from one person.<br>0 = Unable to walk or requires assistance from two or more persons.<br>Assistive devices may be used and recorded (e.g. T-cane, quad cane, orthosis, walker).
Gait	8. Walking speed	0-2	2 = $\geq$ 0.8 m/s ($\leq$ 12.5 s in a 10-m walking test) or a speed sufficient for independent outdoor ambulation.<br>1 = $\geq$ 0.4 m/s and $<$ 0.8 m/s (12.5-25 s in a 10-m walking test) or a speed sufficient for independent indoor ambulation.<br>0 = $<$ 0.4 m/s ($>$ 25 s in a 10-m walking test) or not independent indoors.<br>Note: A comfortable 10-m or 5-m walking test may be used, with a cane or orthosis if needed. If direct measurement is not feasible, speed may be estimated and recorded as an estimate.
Gait	9. Gait abnormality	0-2	2 = No observable deviation from normal gait.<br>1 = Deviation from normal gait is observed in either the stance phase or the swing phase.<br>0 = Deviation from normal gait is observed in both the stance and swing phases.

			Examples of stance-phase deviation include asymmetry of stance time, asymmetry of step length, wide base of support, initial contact with a part of the foot other than the heel, and excessive knee instability. Examples of swing-phase deviation include toe drag, pelvic hiking, and excessive hip abduction or circumduction. Any observable difference from normal gait is regarded as a deviation.
Gait	10. Stair negotiation	0-3	3 = Ascends and descends stairs without using a handrail, with an alternating-step pattern.<br>2 = Ascends and descends stairs using a handrail, with an alternating-step pattern.<br>1 = Ascends and descends stairs using a handrail, with a step-to pattern.<br>0 = Cannot negotiate stairs safely without assistance.<br>Note: Orthoses may be used.

B. Other SPTA Components Analyzed in the Present Study

Component	Item	Score	Scoring criteria
Muscle strength	Ankle dorsiflexion; knee extension; hip flexion	0-3	The more severely affected side is assessed; if there is no clearly more severely affected side, either side may be assessed.<br>3 = Normal strength comparable to a healthy individual.<br>2 = Movement against gravity but weaker than the unaffected side or a healthy individual.<br>1 = Visible movement but unable to move against gravity.<br>0 = No visible movement.
Pain	Pain at rest	0-2	2 = No pain.<br>1 = Pain is perceived but is not intolerable.<br>0 = Intolerable pain.<br>If present, the posture and timing may be recorded (lying, sitting, standing; daytime or nighttime).
Pain	Pain during movement	0-2	2 = No pain.<br>1 = Pain is perceived but is not intolerable.<br>0 = Intolerable pain.<br>If present, the painful movement may be recorded as pain during a specific movement or nonspecific pain during continued movement.
Upper extremity function	Object manipulation in ADL	0-5	One task is selected from feeding, grooming, dressing, or another relevant ADL task, and the most difficult task for the participant is evaluated.<br>5 = Movement comparable to that of a healthy individual.<br>4 = Movement close to that of a healthy individual but slower and with reduced dexterity.<br>3 = Movement differs from healthy movement, is slow, and requires effort.<br>2 = The task can be completed but requires task modification or preparation.<br>1 = The task cannot be completed, but voluntary movement is present.<br>0 = No voluntary movement.
Upper extremity function	Frequency of object manipulation	0-2	Assessed for the task selected in the object-manipulation item.<br>2 = Uses the affected upper limb with approximately the same frequency as before onset.

			1 = Occasionally uses the affected upper limb, but mostly uses only the unaffected side.<br>0 = Does not use the affected upper limb in daily life.<br>If both sides are affected, frequency is judged relative to healthy individuals.
Life-space mobility	Indoor mobility outside the room	Composite score	Assessed for the most recent 4 weeks or since onset. The composite score is calculated as mobility attainment x frequency x assistance.<br>Mobility attainment: 1 = yes; 0 = no.<br>Frequency: 0 = none; 1 = less than once per week; 2 = 1-3 times per week; 3 = 4-7 times per week.<br>Assistance: 0 = does not move to that level; 1 = personal assistance; 1.5 = equipment only; 2 = no assistance or equipment.
Life-space mobility	Outdoor mobility outside the home	Composite score	Assessed for the most recent 4 weeks or since onset. The composite score is calculated as mobility attainment x frequency x assistance.<br>Mobility attainment: 1 = yes; 0 = no.<br>Frequency: 0 = none; 1 = less than once per week; 2 = 1-3 times per week; 3 = 4-7 times per week.<br>Assistance: 0 = does not move to that level; 1 = personal assistance; 1.5 = equipment only; 2 = no assistance or equipment.

C. Note on external comparator items

For construct validity analysis, the manuscript also recorded the Functional Independence Measure (FIM) motor items as external comparator measures. These items are not reproduced here because the FIM is an established published instrument and was not part of the newly developed MQS/SPTA scoring sheet.

Note. This appendix is not a full literal translation of the complete original form. Rather, it is a manuscript-specific English summary of the items and scoring rules analyzed in the present study, prepared to improve clarity and reproducibility for readers of the Journal of Rehabilitation Medicine.
